# Effective fraction of *Bletilla striata* reduces the inflammatory cytokine production induced by water and organic extracts of airborne fine particulate matter (PM_2.5_) in vitro

**DOI:** 10.1186/s12906-019-2790-3

**Published:** 2019-12-16

**Authors:** Yu-Yao Zu, Quan-Fang Liu, Shu-Xin Tian, Li-Xia Jin, Fu-Sheng Jiang, Mei-Ya Li, Bing-Qi Zhu, Zhi-Shan Ding

**Affiliations:** 1 College of Life Science, Zhejiang Chinese Medical University, Zhejiang, 310053 Hangzhou China; 2 College of Medical Technology, Zhejiang Chinese Medical University, Zhejiang, 310053 Hangzhou China; 3 Academy of Chinese Medical Sciences, Zhejiang Chinese Medical University, Hangzhou, 310053 China

**Keywords:** *Bletilla striata* (Thunb.) Rchb.F., PM_2.5_ extracts, Macrophage, Inflammation, NF-κB/MAPK pathway

## Abstract

**Background:**

*Bletilla striata* is a traditional Chinese medicine used to treat hemorrhage, scald, gastric ulcer, pulmonary diseases and inflammations. In this study, we investigated bioactivity of the effective fraction of *B. striata* (EFB) in reducing the inflammatory cytokine production induced by water or organic extracts of PM_2.5_.

**Methods:**

PM_2.5_ extracts were collected and analyzed by chromatographic system and inductively coupled plasma mass spectrometer. Cell viability was measured using MTS (3-(4,5-dimethylthiazol-2-yl)-5-(3-carboxymethoxyphenyl)-2-(4-sulfophenyl)-2H-tetrazolium) assay, and cell supernatant was analyzed by flow cytometry, ELISA, and qRT-PCR in cultured mouse macrophage cell line RAW264.7 treated with EFB and PM_2.5_ extracts. Expressions of nuclear factor-kappa B (NF-κB) and mitogen-activated protein kinase (MAPK) signaling pathway were measured by Western blot.

**Results:**

PM_2.5_ composition is complex and the toxicity of PM_2.5_ extracts were not noticeable. The treatment of EFB at a wide dose-range of 0–40 μg/mL did not cause significant change of RAW264.7 cell proliferation. EFB pretreatment decreased the inflammatory cytokines in the macrophage. Further analysis showed that EFB significantly attenuated PM_2.5_-induced proinflammatory protein expression and downregulated the levels of phosphorylated NF-κBp65, inhibitor of kappa B (IκB)-α, c-Jun N-terminal kinase (JNK), extracellular signal-regulated kinase (ERK), and p38.

**Conclusions:**

Our study demonstrated the potential effectiveness of *B. striata* extracts for treating PM_2.5_-triggered pulmonary inflammation.

## Background

Airborne fine particulate matter (PM_2.5_) poses a high risk to human health worldwide. Epidemiological studies have shown that exposure to PM_2.5_ is strongly related to chronic bronchitis, asthma, chronic obstructive pulmonary disease (COPD), emphysema, lung cancer, and other respiratory diseases [1–3]. The components of PM_2.5_ are complicated and they have been reported to include water-soluble inorganics, toxic metals, polycyclic aromatic hydrocarbons (PAHs), and bacterial endotoxins [4]. Pulmonary macrophage-mediated inflammation plays a vital role in PM_2.5_-induced pulmonary dysfunction [5, 6]. The transcription nuclear factor-kappa B (NF-κB) is closely associated with inflammatory cytokine production in pulmonary macrophages [7]. The NF-κB heterodimer involving Rel (p65) and p50 proteins is a latent cytoplasmic factor that can be found in the cytosol complexed with the inhibitory protein inhibitor of kappa B (IκB)-α [8]. Upon stimulation, IκBα dissociates from the heterodimer, which enables the heterodimer to translocate into the nucleus where it binds to specific DNA sequences, e.g., the interleukin (IL)-6 and tumor necrosis factor (TNF)-α promoters. In addition, mitogen-activated protein kinase (MAPK), including extracellular signal-regulated kinase (ERK), c-Jun N-terminal kinase (JNK), and p38 kinase, are also activated in PM_2.5_-treated macrophages [9–11]. Activated MAPK can upregulate inflammatory cytokine expression via phosphorylation of the downstream transcription factor, activator protein-1 (AP-1). Therefore, the NF-κB and MAPK pathways are key targets for the treatment of PM_2.5_-induced pulmonary inflammation and dysfunction [12].

*Bletilla striata* (Thunb.) Rchb. f., which is a traditional Chinese medicine, has been widely used for its pulmonary protective, hemostasis, analgesic, antiulcer, anti-fibrosis, and anti-inflammatory effects [13–15]. Numerous reports have demonstrated the exitance of various chemical components of *B. striata*, including bibenzyls, phenanthrenes, polysaccharides, anthocyanidins, dihydrophenanthrenes, steroids, triterpenes, and glycosides [16], which exhibit a variety of biological and pharmacological activities. For instance, *B. striata* polysaccharide was reported to reduce the levels of pro-inflammatory cytokines and suppress MAPK/NF-κB signaling pathway activity in rats with gastric ulcer induced by ethanol [17]. In our previous study, we used to carried out the utilization probability of the fibrous root part of *B. striata*, studied the pharmacological activities of *B. striata* extract [18], exploring the effects of antiviral and antibacterial activity, purifying the total effective fraction of *B. striata* (EFB) and efficacy components [19], and obtaining it’s extracts using a liquid chromatography silica gel column and semipreparative liquid chromatography [20]. At the same time, experimental results obtained in vitro cell system analysis confirmed the antioxidant activity of *B. striata*, which can induce HepG2 cells apoptosis in a dose-dependent manner [21]. Studies have shown that PM_2.5_ could trigger pulmonary inflammation and oxidative stress, which result in pulmonary fibrosis [22, 23], our previous experiments have documented that the ethanol extract of *B. striata* exhibited a variety of therapeutic effects including anti-inflammatory, and anti-fibrosis by significantly down regulated serum levels of IL-1β, TNF-α, transforming growth factor-(TGF-β) and other inflammatory factors [24, 25], and are more effective than the polysaccharide of *B. striata* [26]. Besides this, a recent study by Luo [27] showed that the polysaccharide of *B. striata* decreased the inflammatory cytokine levels of IL-6 and TNF-α to protect IEC-18 cells from lipopolysaccharide (LPS)-induced injury. Hence, we speculated that the extract of *B. striata* can impact PM_2.5_-induced injury.

In recent years, a large number of scientific studies, including clinical trials, have been conducted in the field of traditional Chinese medicine, and it has been found that *B. striata* and extracts have anti-inflammatory and anti-oxidative effects [28, 29]. The effect of anti-PM_2.5_ induced inflammation by *B. striata* has not been reported. 2,7-dihydroxy-4-methoxy-9,10-dihydrophenanthrene (Coelonin), as one of the main active components from *B. striata* total ethanol-extract (BTE) was separated and can significantly down regulated IL-1β and IL-6 expression on LPS-induced RAW264.7 cells [25]. Therefore, Coelonin may be one of the main active components contributing to the anti-inflammatory of *B. striata*. The study was to explore the protective effects of BTE and Coelonin on PM_2.5_-induced inflammatory cytokine expression in macrophages and investigate the underlying mechanism of the prevention and treatment effects of *B. striata* regarding PM_2.5_-related inflammatory disease.

## Methods

### Preparation and chemical analyses of PM_2.5_

The particles were deposited on 203 mm × 254 mm glass fiber filters purchased from Whatman (Little Chalfont, Buckinghamshire, UK), which had been prebaked at 300 °C for 5 h before use. Samples of PM_2.5_ were collected on glass fiber filters using a Thermo Anderson G-2.5 large-volume sampler (Waltham, MA, USA) with a flow rate of 1.13 m^3^/min. The sampling location was set on the rooftop of laboratory building 4 at the Zhejiang Chinese Medical University, which was 20 m higher above the ground, without obvious nearby source of pollution, from October 1, 2016 to March 3, 2017.

For water extraction, small pieces of the glass fiber filter (0.5 × 0.5 cm) were cut and immersed in ultrapure water. For organic extraction, small pieces of the glass fiber filter (0.5 × 0.5 cm) were cut and immersed in dichloromethane. These samples were sonicated for 6 × 30 min. Water extracts were filtered through 0.45 μm filters and freeze-dried, while organic extracts were rotary-evaporated, and stored at − 80 °C for later use. Water extracts of PM_2.5_ (WEP) were then diluted to 10 mg/mL using phosphate-buffered saline (PBS) buffer, while organic extracts of PM_2.5_ (OEP) were diluted in dimethyl sulfoxide (DMSO)/sterile PBS (with a final DMSO concentration < 0.5%). The stock solution was sonicated for 45 min before the experiment and then further diluted to the desired concentration with Dulbecco’s Modified Eagle Medium (DMEM; CellMax, Beijing, China).

Water-soluble inorganic ions, including Na^+^, K^+^, Mg^2+^, Ca^2+^, NH_4_^+^, SO_4_^2−^, NO_3_^−^, and Cl^−^ in the PM_2.5_ water extract was analyzed using an ICS-2000 ion chromatography system (Dionex, USA) equipped with an AS3000 autosampler. Ion standard materials were purchased from the National Standards Center (Beijing, China). A total of 20 kinds of soluble metals were detected in the water extracts using an inductively coupled plasma mass spectrometer (ICP-MS; Thermo X series; Thermo Fisher Scientific, Waltham, MA, USA). The analytical model involved a full quantitative analysis; the oxide level was < 2%; the double charge formation was < 3%. The samples were analyzed under the above optimized conditions, and rhenium were used as internal standards. A 7890A-5975C gas chromatography-mass spectrometer (GC-MS; Agilent Technologies, Santa Clara, CA, USA) equipped with an electron ionization (EI) ion source was used to conduct PAHs analysis in the organic extracts [30]. Blank filters were analyzed to check for chemical contamination during the field and laboratory operations.

### Preparation of *B. striata* effective fraction

*B. striata* was collected from Meichuan Town in Hubei Province, People’s Republic of China, and authenticated as a purple orchid with medicinal properties by Prof. Zhi-Shan Ding (one of the authors). A voucher specimen (LA-20161103) has already been deposited in Zhejiang Chinese Medical University. The dry tubers of *B. striata* were processed, smashed, and filtered through a 40-mesh sieve. 100 g powder was refluxed and extracted three times with 1 L of 95% ethanol. The filtrate was allowed to cool, filtered, and dried in the reduced pressure distillation. The obtained semi-solid *B. striata* total ethanol-extract (BTE) was stored at − 20 °C until used.

The BTE was loaded onto a polyamide resin column and washed with distilled water, followed by elution with 40% (v/v) ethanol. The active fraction eluted with 40% ethanol in water were dried in vacuum and analyzed using a Dionex Ultimate3000 high-performance liquid chromatography (HPLC, USA), then, separation and purification were carried out by Dionex Ultimate3000 semi-preparative high-performance liquid chromatography (semi-preparative HPLC, USA). A Welch Ultimate XB-C_18_ column (10 × 250 mm, 10 μm) was used with the column temperature set at 30 °C, and elution solvents were acetonitrile with 0.1% formic acid. Analytes were monitored at 280 nm. The peaks with a retention time of 12.5~15.5 min was collected to obtain the extract of BTE, and analyzed by HPLC fingerprints and SYNAPT G2-Si HD Mass Spectrometer (Waters, USA) to identify the extract as Coelonin (Additional file 1). 5 mg/mL BTE and Coelonin were diluted using DMSO, which were further diluted to desired concentrations with DMEM in experiment (with a final DMSO concentration < 0.5%).

### Cell culture

The mouse macrophage cell line RAW264.7 was purchased from Shanghai Institutes for Life Science, Chinese Academy of Sciences (Shanghai, China). The RAW264.7 cells were cultured in DMEM containing 10% fetal bovine serum (FBS), which was obtained from CellMax, supplemented with 100 units/mL penicillin and 100 mg/mL streptomycin at 37 °C in a humidified 5% CO_2_ atmosphere. The cells were passaged every 2–3 days, after reaching 70–80% confluency.

### Cell viability analysis

RAW264.7 cells were plated in a 96-well plate at a density of 5 × 10^4^ cells per well. After overnight growth, the culture medium was removed. To assess the effects on cell viability, 100 μL of cell culture medium containing either PM_2.5_ water or organic extracts at 12.5, 25, 50, 100, and 200 μg/mL were added to each well, and either BTE or Coelonin at 1.25, 2.5, 5, 10, 20, and 40 μg/mL were added. After 24 h of incubation, 20 μL MTS (3-(4,5-dimethylthiazol-2-yl)-5-(3-carboxymethoxyphenyl)-2-(4-sulfophenyl)-2H-tetrazolium); Promega, Madison, WI, USA) was added to each well for 2 h of incubation at 37 °C in a humidified 5% CO_2_ incubator. The absorbance at 490 nm of the formazan was assessed using a BioTek Epoch2 microplate reader (Winooski, VT, USA).

### Measurement of TNF-α, IL-6, and monocyte chemoattractant protein (MCP)-1

RAW264.7 cells were plated in a 96-well plate at a density of 1 × 10^5^ cells per well in 100 μL of culture media and incubated for 12 h. Subsequently, the differentiated RAW264.7 cells were treated with PM_2.5_ at different concentrations of 12.5, 25, 50, 100, and 200 μg/mL for an additional 12 and 24 h. The supernatant of the PM_2.5_ extract-treated cells was collected to assess TNF-α secretion using an enzyme-linked immunosorbent assay (ELISA) obtained from eBioscience (San Diego, CA, USA).

Further, other cells were pretreated with BTE at 0, 2.5, 5, 10, and Coelonin at 0, 1.25, 2.5, 5, and 10 μg/mL for 2 h before treating with 200 μg/mL PM_2.5_ water extract, in another group, same treatments by BTE and Coelonin were repeated and followed by treating with 100 μg/mL organic extract. After 18 h treatment, the supernatant of these cells was then collected for the analysis of TNF-α, IL-6, and MCP-1 secretion using an ELISA and a BD Biosciences cytometric bead array assay (San Jose, CA, USA). Each immunoassay was performed in accordance with the manufacturer’s instructions.

### Quantitative real-time PCR (qRT-PCR) analysis

Total RNA was extracted with RNAiso Plus (Takara, Tokyo, Japan) according to the manufacturer’s instruction, and dissolved in 30 μL diethylpyrocarbonate (DEPC)-H_2_O. The total RNA was reverse-transcribed to cDNA using a PrimeScript™ RT Master Mix kit (Takara, Shiga, Japan). Relative expression levels of IL-6 and TNF-α were quantified using SYBR® Premix Ex Tap™ II (Takara, Shiga, Japan). All primer sequences (Sangon Biotech, Shanghai, China) are listed in Table 1. PCR cycle were conducted according to the following conditions: initial denaturation at 95 °C for 2 min, 40 cycles of denaturation at 95 °C for 15 s, annealing at 60 °C for 20 s, and extension at 72 °C for 15 s. Relative gene expression levels, obtained based on reverse transcription qRT-PCR, were calculated using the 2^-ΔΔCt^ method following normalization to glyceraldehyde 3-phosphate dehydrogenase (GAPDH).

**Table 1:** Primer sequences used for qRT-PCR analysis

Gene Name	Primer Sequence (5′ - > 3′)
GAPDH	Foward CATCACTGCCACCCAGAAGACT
	Reverse GACACATTGGGGGTAGGAACAC
TNF-α	Foward CGAGTGACAAGCCTGTAGCCC
	Reverse GGGCAGCCTTGTCCCTTGA
IL-6	Foward AGTTGCCTTCTTGGGACTGA
	Reverse TTCTGCAAGTGCATCATCGT

### Western blot analysis

For the western blot analysis, the PM_2.5_ water extract at a concentration of 200 μg/mL was used and the PM_2.5_ organic extract at a concentration of 100 μg/mL was used. Total proteins were isolated using a protein extraction kit (Beyotime, Shanghai, China). Harvested proteins were denatured at 95 °C for 10 min, separated by sodium dodecyl sulfate polyacrylamide gel electrophoresis (SDS-PAGE), and then transferred onto a polyvinylidene fluoride (PVDF) membrane (Millipore Corp., USA). The membranes were blocked with 5% bovine serum albumin (BSA; Biosharp, Hefei, China) for 1 h. They were subsequently probed at 4 °C overnight with the primary antibodies, comprising anti-β-actin (110000); anti-IκBα (1:1000), anti-phospho-IκBα (1:1000), anti-NF-κB p65 (15000), anti-phospho-NF-κBp65 (1:2000), anti-p38 (1:2000), anti-phospho-p38 (1:1000), anti-JNK1 + JNK2 + JNK3 (1:2000), anti-phospho-JNK1 + JNK2 + JNK3 (1:1000), anti-ERK1 + ERK2 (1:2000) and anti-phospho-ERK1 + ERK2 (1,1000) purchased from Abcam (Cambridge, MA, UK). Subsequently, the membranes were incubated for 1 h with a horseradish peroxidase (HRP)-conjugated secondary antibody (15,000, Jackson ImmunoResearch Laboratories, West Grove, PA, USA) at room temperature. Enhanced chemiluminescence (ECL) substrate (Perkin Elmer, Waltham, MA, USA) was added to detect the proteins using a chemiluminescence imaging system (C-DiGit Blot Scanner, LI-COR, USA).

### Statistical analysis

All data are presented as mean ± standard deviation. A statistical comparison of the between-group differences was performed using one-way analysis of variance (ANOVA) in SPSS17.0 software (SPSS, Inc., Chicago, IL, USA). *P* values < 0.05 were considered significant.

## Results

### Characterization of major PM_2.5_ components

The concentrations of metal elements in the PM_2.5_ water extract, and the concentrations of PAHs in the PM_2.5_ organic extract were measured, and results are shown in Table 2. The composition of PM_2.5_ in the atmosphere is very complex due to many factors, such as the combustion source, industry pollution, climate, and season [31].

**Table 2:** Concentrations of elements in water and PAHs in organic extracts both at levels of 100 μg/mL

Content (μg/mL)	Metal elements in water extracts	Content (μg/mL)	PAHs in organic extracts
Sodium (Na) Magnesium (Mg) Phosphorous(P) Kalium(K) Lead (Pb) Calcium (Ca) Aluminium (Al) Titanium (Ti) Vanadium(V) Chromium (Cr) Manganese (Mn) Iron (Fe) Cobalt (Co) Nickel (Ni) Copper (Cu) Zinc (Zn) Arsenic (As) Cadmium (Cd) Barium (Ba) Thallium (TI)	5808.89 965.45 155.08 2460.90 9.20 7136.49 72.11 0.96 4.91 3.54 81.79 102.02 0.94 4.23 39.38 1051.85 31.87 4.05 40.04 2.97	2-Methylfluoranthene 2-Methylphenanthrene 1-Methylphenanthrene 1-Methylpyrene Perylene Benzo(e)pyrene Dibenzo(a, h)anthracene Fluoranthene Benzo(a)anthracene Benzo(c)phenanthrene Phenanthrene Pyrene Naphthalene FIuorene Chrysene Benzo(b,j)fluoranthene Benzo(a)pyrene Benzo(k)fluoranthene Acenaphthylene Anthracene	0.16 0.07 0.09 0.17 0.26 0.80 0.13 1.24 0.62 0.15 0.48 1.12 0.05 0.04 1.04 2.66 0.99 0.54 0.03 0.03

### Cell viability

**Fig. 1:**
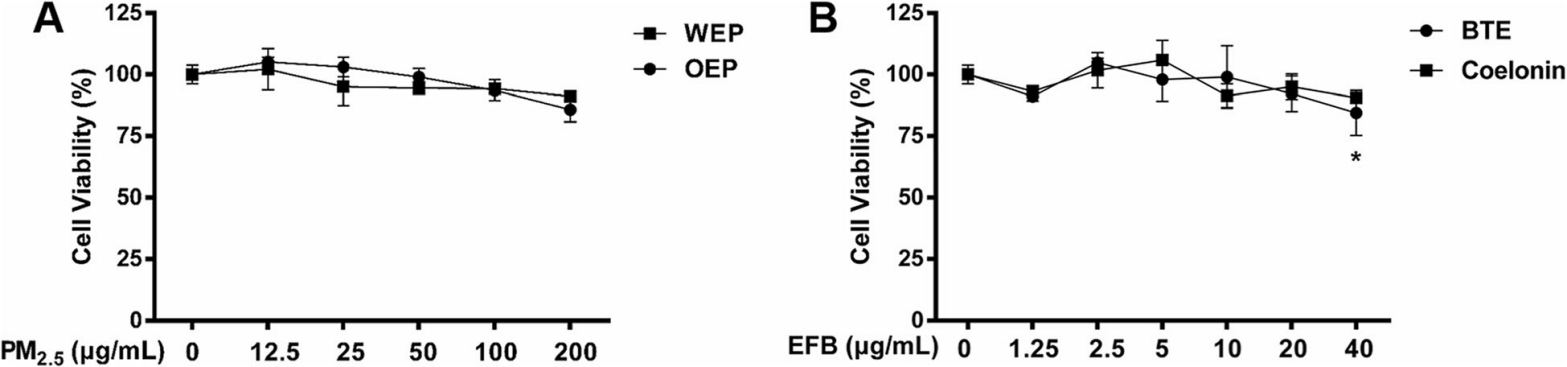
Cell viability of RAW264.7 cells after exposure to PM_2.5_ extracts and EFB. (**a**) Cell viability after exposure to WEP and OEP. (**b**) Cell viability after exposure to BTE and Coelonin. * < 0.05 vs. control

The viability of RAW264.7 cells exposed to PM_2.5_ water and organic extracts was evaluated using MTS assays. After 24 h exposure of RAW264.7 cells to a wide dose range of PM_2.5_ water and organic extracts from12.5 μg/mL to 200 μg/mL, the cell viability slightly decreased at the high doses (100 and 200 μg/mL) as presented in Fig. 1 a, indicating that the toxicity of PM_2.5_ was not noticeable. Figure 1 b shows that BTE and Coelonin had no significant effects on the growth of RAW264.7 cells in the concentrations range of 0–20 μg/mL, but significantly inhibitions were observed at 40 μg/mL. Therefore, based on MTS assay and previous study [25], the concentrations of BTE used in the following experiments were 2.5, 5, 10, and 20 μg/mL, while the concentrations of Coelonin used in the following experiments were 1.25, 2.5, 5, and 10 μg/mL.

### Effective fraction of *B. striata* inhibits PM_2.5_-induced inflammatory cytokine expression in RAW264.7 cells

**Fig. 2:**
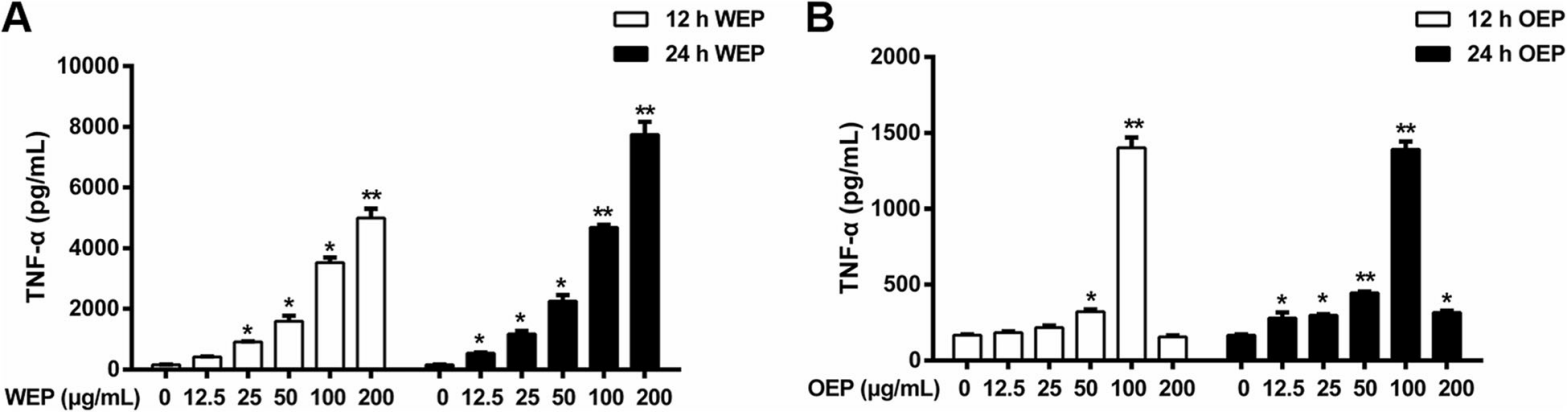
TNF-α expression after exposure PM_2.5_ extracts. TNF-α expression in RAW264.7 cells treated for 12 and 24 h with (**a**) WEP and (**b**) OEP. * < 0.05, ** < 0.01 vs. control

Inflammation played a major role in cell damage caused by PM_2.5_. In vitro, PM_2.5_ has been shown to induce inflammatory responses in various human and animal cells with time and dose dependent increases in gene expression [32], and it was found to result in increased macrophage infiltration and significantly upregulated TNF-α and IL-6 levels [33]. In the current study, TNF-α secretion by RAW264.7 cells exposed to PM_2.5_ extracts at 12.5, 25, 50, 100, and 200 μg/mL for 12 and 24 h, were evaluated using ELISA. Figure 2 a indicates that TNF-α significantly increased in the cells treated by PM_2.5_ water extract in a concentration-dependent manner after comparing with the untreated cells. Figure 2 b illustrates that TNF-α secretion peaked in cells treated with 100 μg/mL PM_2.5_ organic extract. A significant injury was found in treated cells with 200 μg/mL WEP or 100 μg/mL OEP, and the dose was used in further experiments.

**Fig. 3:**
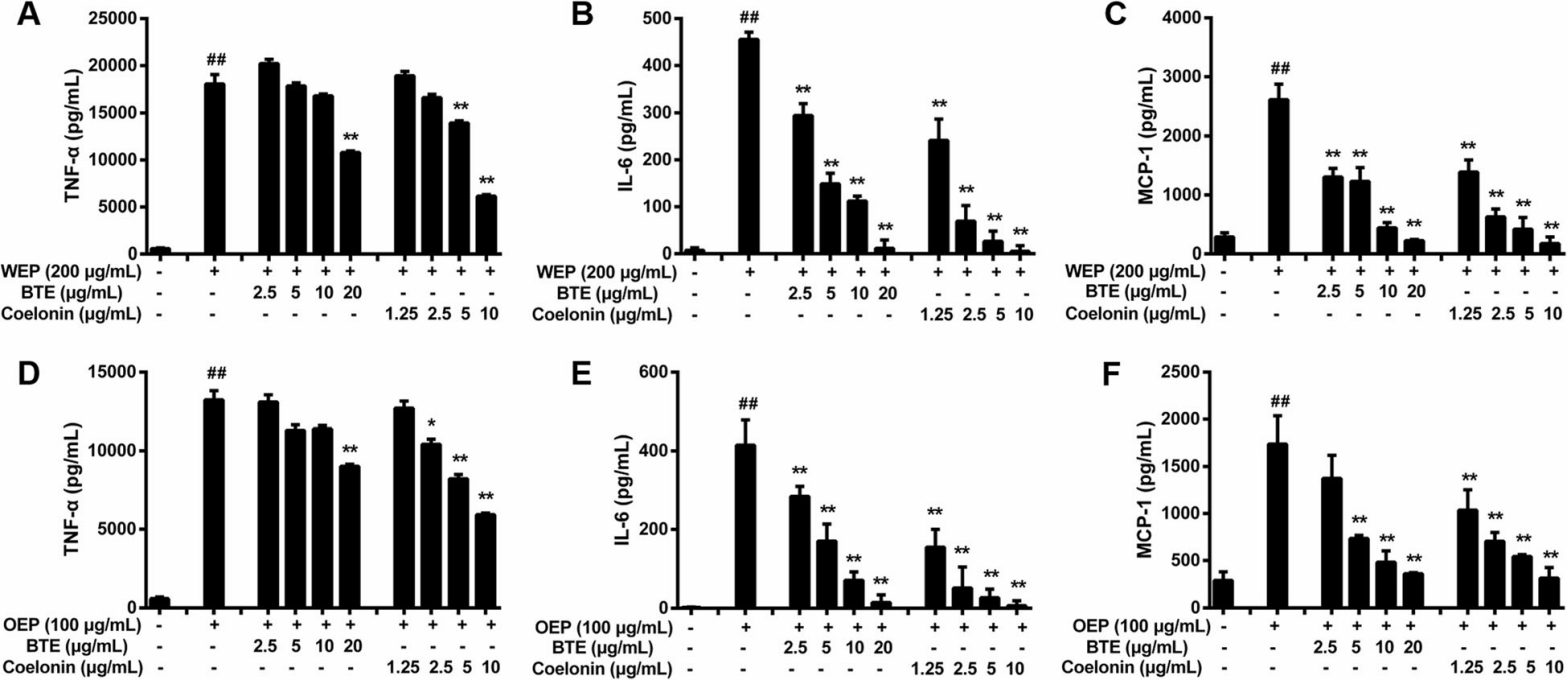
EFB pretreatment prevented PM_2.5_ extracts induced proinflammatory cytokines secretion in RAW264.7 cells. TNF-α (**a**), IL-6 (**b**), and MCP-1(**c**) synthesis in RAW264.7 cells exposed to 200 μg/mL WEP, which was attenuated by BTE and Coelonin. # < 0.05, ## < 0.01 vs. WEP-only group. TNF-α (**d**), IL-6 (**e**), and MCP-1 (**f**) synthesis in RAW264.7 cells exposed to 100 μg/mL OEP, which was attenuated by BTE and Coelonin. # < 0.05, ## < 0.01 vs. OEP-only group

BTE and Coelonin were used to treat RAW264.7 cells and followed by exposure to 200 μg/mL PM_2.5_ water extract or 100 μg/mL PM_2.5_ organic extract. The IL-6, TNF-α, and MCP-1 levels were elevated after exposure to PM_2.5_ extracts. The ability of BTE and Coelonin to inhibit TNF-α, IL-6, and MCP-1 secretion induced by PM_2.5_ water or organic extract is illustrated in Fig. 3. In particular, the maximum declines in TNF-α, IL-6, and MCP-1 (compared to the PM_2.5_ extract-only group) occurred for PM_2.5_ water and organic extract-treated cells treated with 20 μg/mL BTE and 10 μg/mL Coelonin (the highest doses tested) (Fig. 3 b,c). For both of the PM_2.5_ water and organic extract-treated cells, the IL-6 and MCP-1 levels decreased in a dose-dependent manner when cells were treated with BTE or Coelonin (Fig. 3e–f). These results indicate that BTE or Coelonin pretreatment at a certain concentration range could reduce TNF-α, IL-6 and MCP-1 secretion in cell supernatants.

### EFB inhibits mRNA expression of inflammatory cytokines

**Fig. 4:**
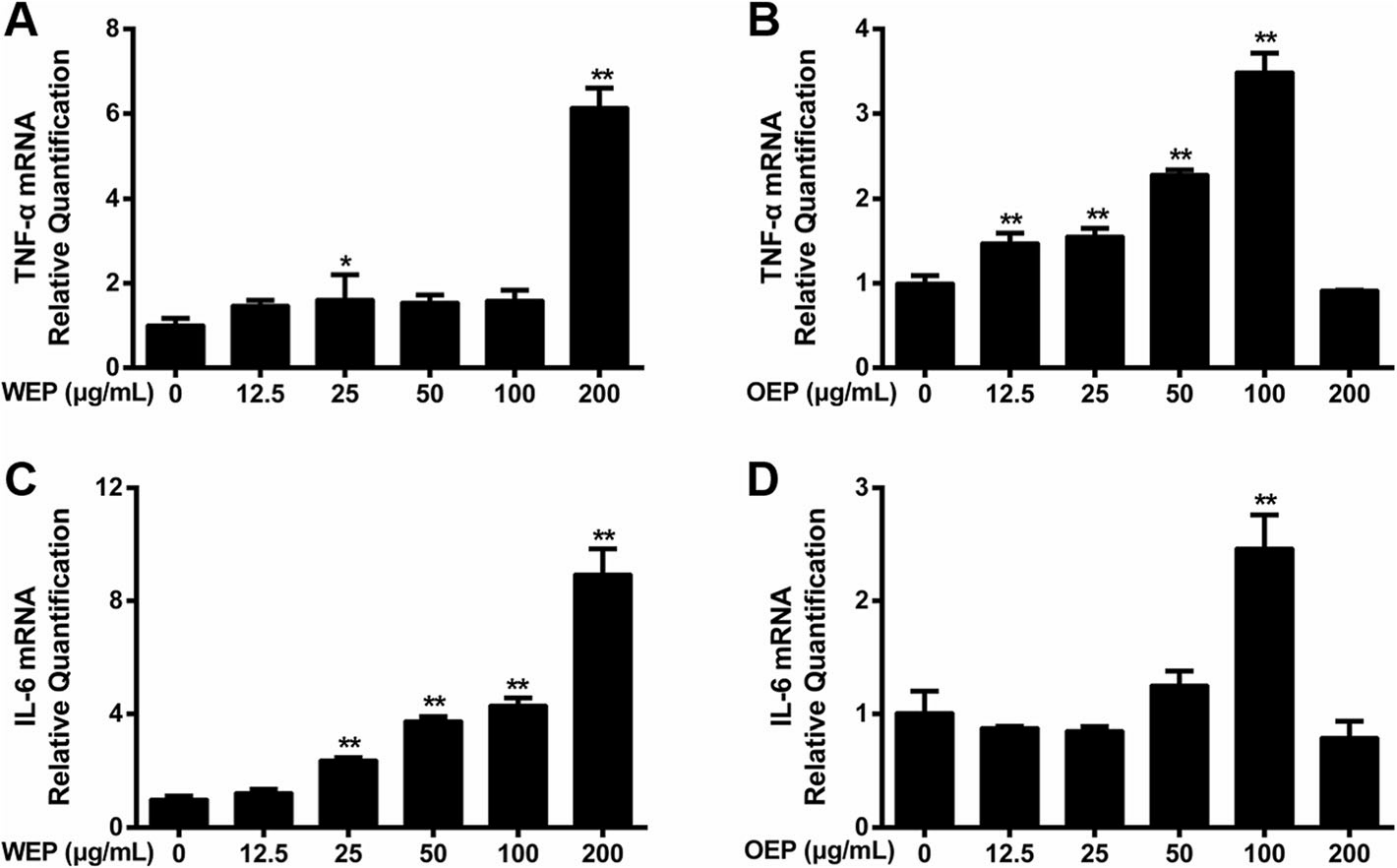
Effects of PM_2.5_ extracts on the mRNA expression of inflammatory cytokines in RAW264.7 cells. After 18 h, mRNA expression of (**a**) TNF-α and (**b**) IL-6 in WEP (200 μg/mL)-treated RAW264.7 cells and of (**c**) TNF-α and (d) IL-6 in OEP (100 μg/mL)-treated RAW264.7 cells. * < 0.05, ** < 0.01 vs. control.

Compared with the untreated RAW264.7 cells, there were obvious changes in TNF-α and IL-6 mRNA expression after the exposure of RAW264.7 cells to PM_2.5_ water and organic extracts (Fig. 4). Regarding the PM_2.5_ water extract, TNF-α and IL-6 mRNA expression was increased highest by 200 μg/mL (Fig. 4a and c). Regarding the PM_2.5_ organic extract, TNF-α and IL-6 mRNA expression was increased highest by 100 μg/mL (Fig. 4b and d).

**Fig. 5:**
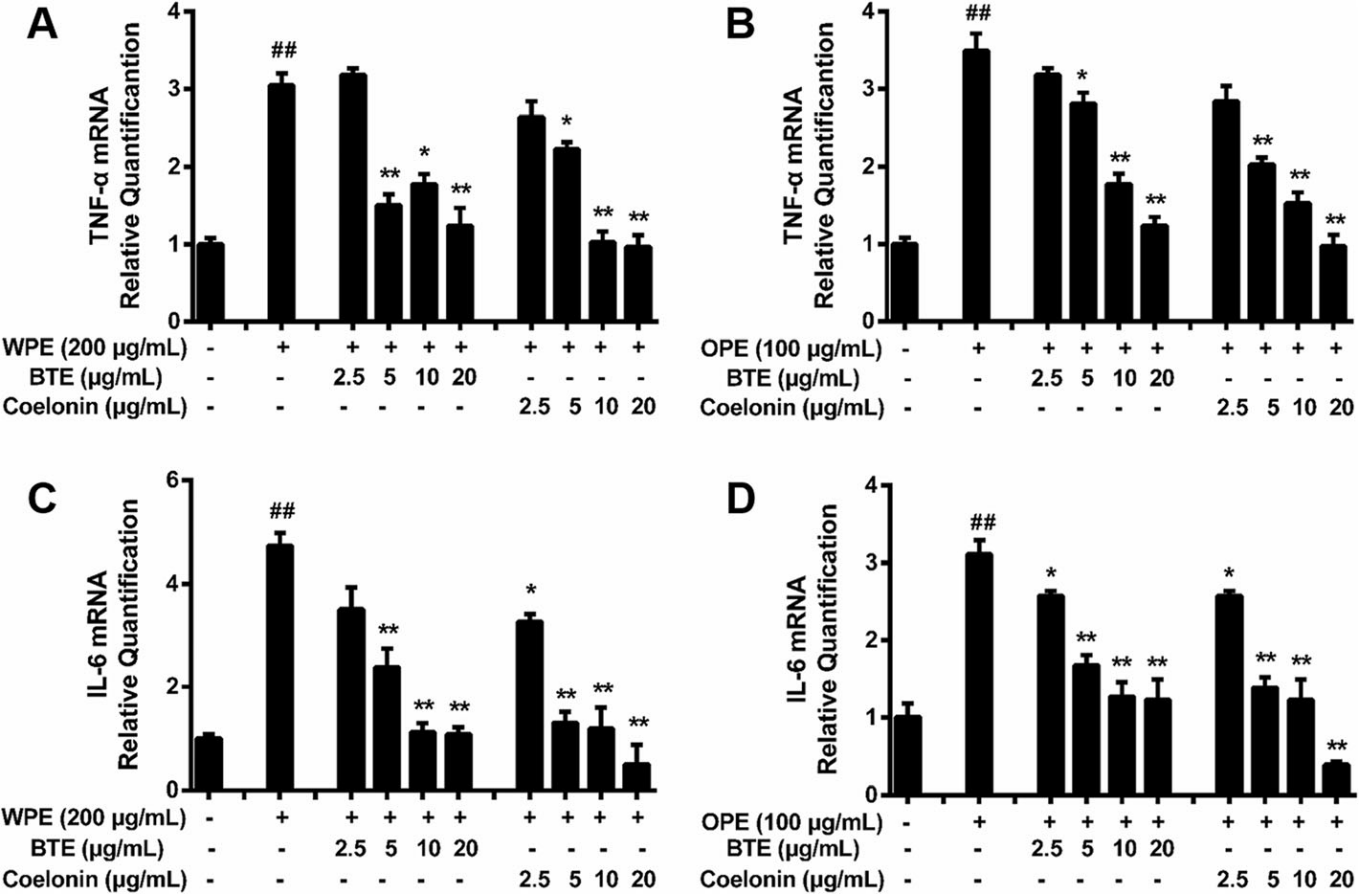
EFB inhibited the effects of PM_2.5_ extracts on the mRNA expression of inflammatory cytokines.mRNA expression of (**a**) TNF-α and (**b**) IL-6 in WEP (200 μg/mL)-treated RAW264.7 cells and of (**c**) TNF-α and (**d**) IL-6 in OEP (100 μg/mL)-treated RAW264.7 cells, which was attenuated by BTE and Coelonin. # < 0.05, ## < 0.01 vs. control. * < 0.05, ** < 0.01 vs. model (PM_2.5_ extract-only group)

BTE (5, 10, and 20 μg/mL) and Coelonin (2.5, 5, and 10 μg/mL) significantly decreased the TNF-α mRNA expression induced by PM_2.5_ water or organic extract (Fig. 5A, B). In addition, BTE (2.5, 5, 10, and 20 μg/mL) and Coelonin (1.25, 2.5, 5, and 10 μg/mL) significantly decreased the IL-6 mRNA expression induced by PM_2.5_ organic extracts in a dose-dependent manner (Fig. 5D), and the results were the same for the PM_2.5_ water extract-treated cells, except that 2.5 μg/mL BTE had no effect (Fig. 5c). BTE or Coelonin reduced mRNA expression and protein synthesis to varying degrees. Briefly, qRT-PCR analysis showed that also performed to determine the protective effect of BTE or Coelonin in cultured mouse macrophage cell line RAW264.7 against PM_2.5_ water or organic extract treatment and the data were generally consistent with above results (Fig. 3 a, b, d and e).

### EFB inhibited the NF-κB/MAPK pathway in PM_2.5_ extract-treated RAW264.7 cells

**Fig. 6:**
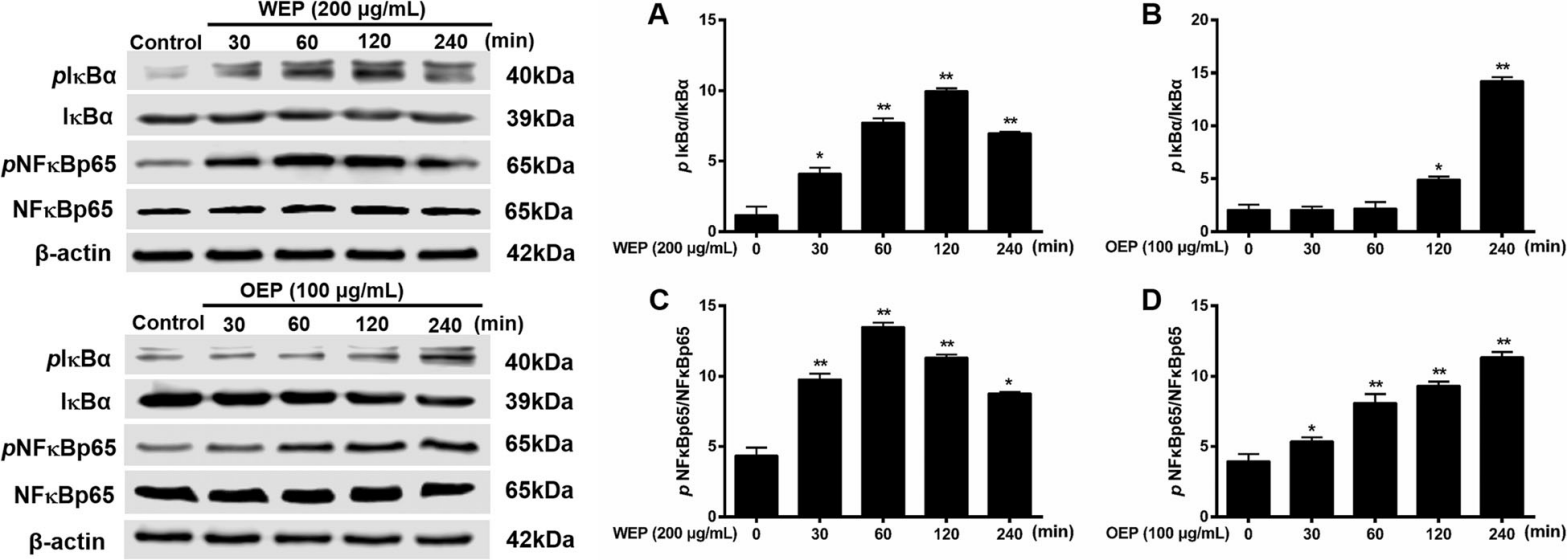
NF-κB pathway activation in RAW264.7 cells after exposure to PM_2.5_ extracts. Protein synthesis in RAW264.7 cells exposed to 200 μg/mL WEP or 100 μg/mL OEP at different time points. * < 0.05, ** < 0.01 vs. control

**Fig. 7:**
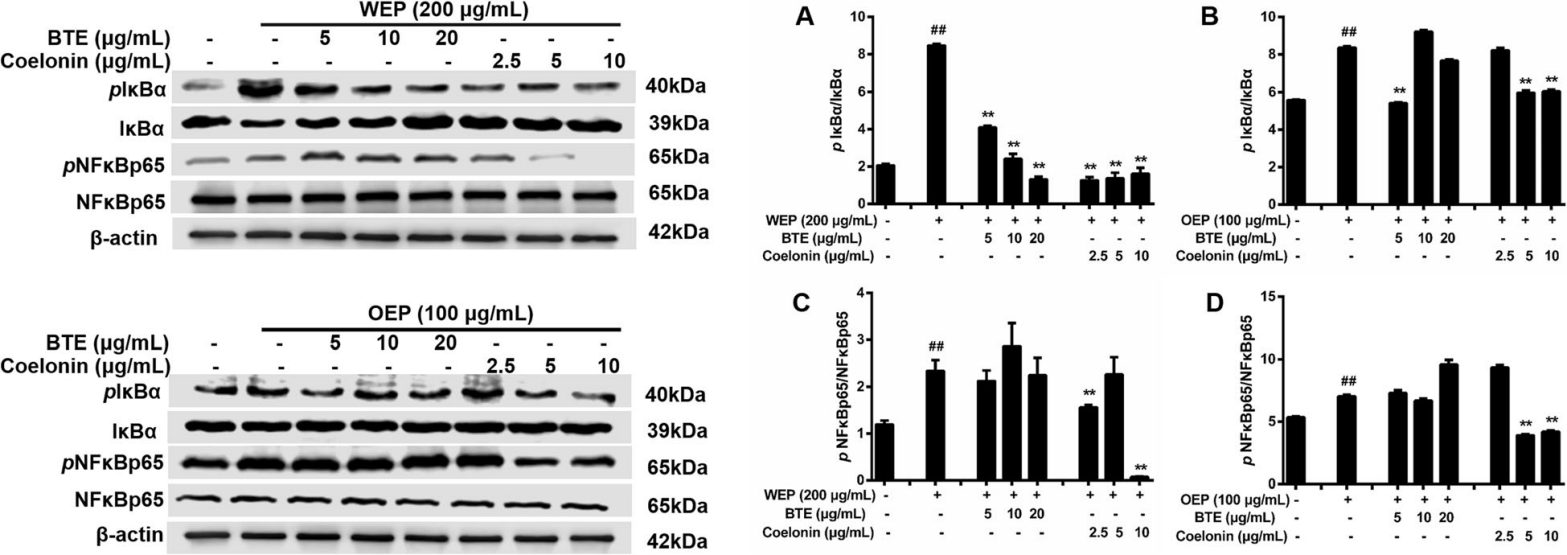
EFB pretreatment inhibited PM_2.5_ extracts evoked activation of NF-κB pathway in RAW264.7 cells. Protein synthesis in RAW264.7 cells exposed to 200 μg/mL WEP or 100 μg/mL OEP, which was attenuated by BTE and Coelonin. # < 0.05, ## < 0.01 vs. control. * < 0.05, ** < 0.01 vs. model (PM_2.5_ extract-only group).

Based on previous literature [34], NF-κB pathway activation in RAW264.7 cells were assessed based on NF-κB and IκBα phosphorylation. NF-κB is a transcription factor that has a crucial role in inflammation, which can with MAPK cascade and provoke to tissue inflammatory damages [35]. In Figs. 6 and 7, we propose a molecular mechanism for the inflammatory cytokine secretion induced by PM_2.5_ extracts. IκBα, NF-κBp65, ERK, JNK, and p38 phosphorylation were significantly increased in PM_2.5_ water extract (200 μg/mL) and organic extract (100 μg/mL)-treated cells compared with untreated cells, and expression level was related to PM_2.5_ extracts treatment time.

**Fig. 8:**
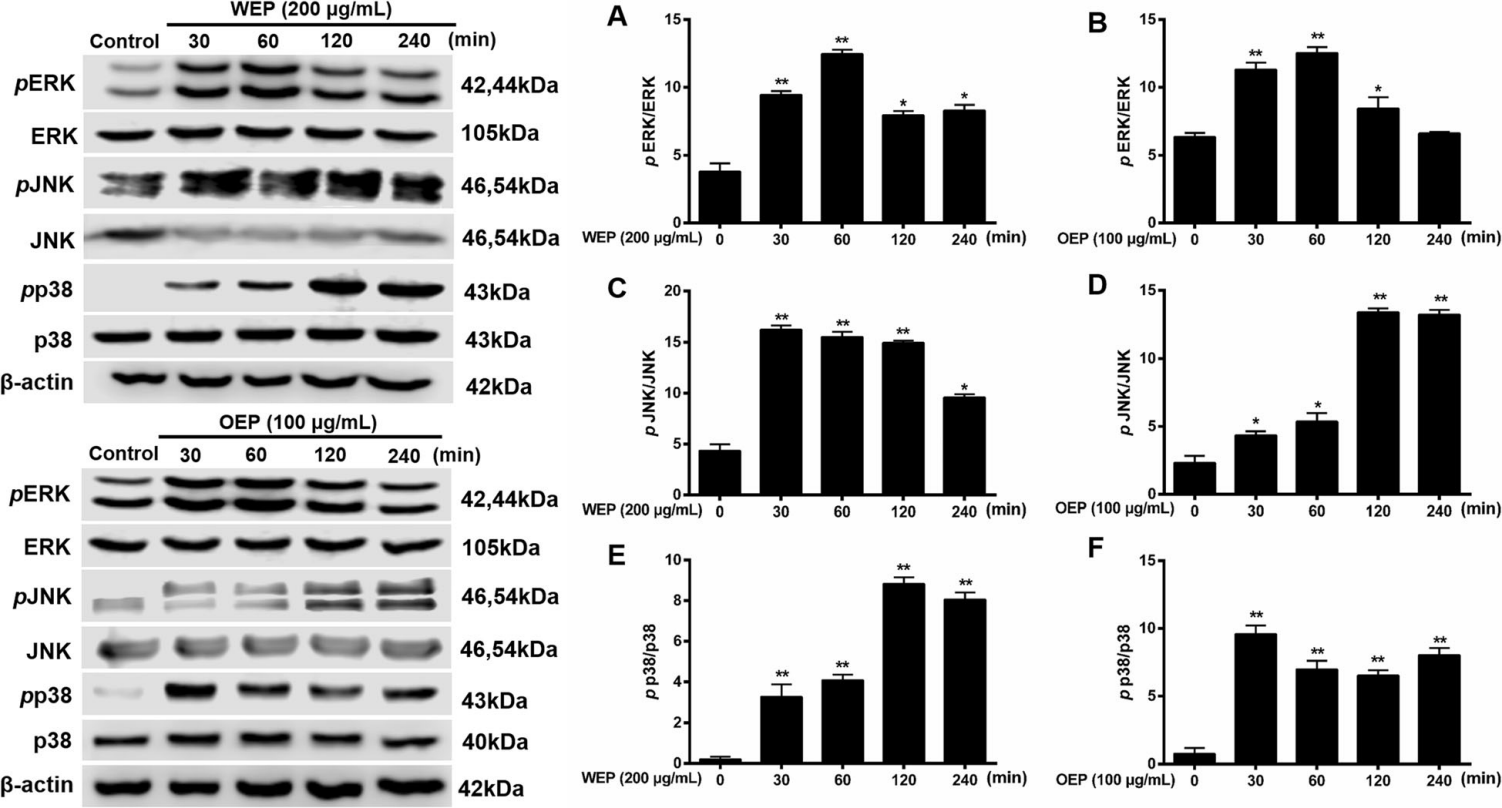
MAPK pathway activation in RAW264.7 cells after exposure to PM_2.5_ extracts. Protein synthesis in RAW264.7 cells exposed to 200 μg/mL WEP or 100 μg/mL OEP at different time points. * < 0.05, ** < 0.01 vs. control.

**Fig. 9:**
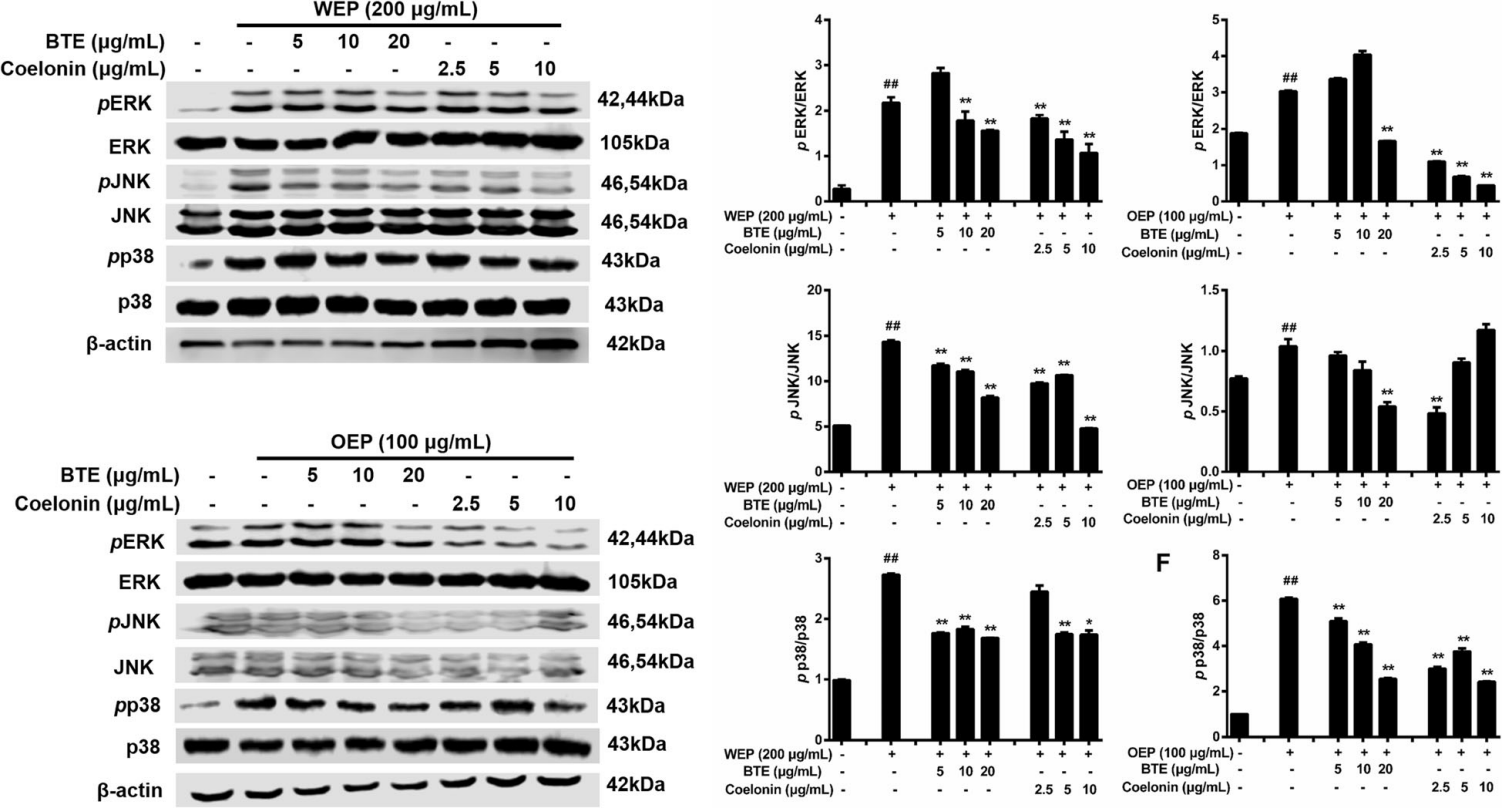
EFB pretreatment inhibited PM_2.5_ extracts evoked activation of MAPK pathway in RAW264.7 cells. Protein synthesis in RAW264.7 cells exposed to 200 μg/mL WEP or 100 μg/mL OEP, which was attenuated by BTE and Coelonin. # < 0.05, ## < 0.01 vs. control. * < 0.05, ** < 0.01 vs. model (PM_2.5_ extract-only group)

To clarify how PM_2.5_ extracts activated NF-κB and MAPK pathway, the mechanistic studies were conducted. BTE and Coelonin acted differently on the pathway activated by the PM_2.5_ water and organic extracts, but both significantly attenuated PM_2.5_ extracts induced phosphorylation. Specifically, the levels of NF-κB family protein were significantly elevated after the treatment of PM_2.5_ extracts, and decreased after the treatment of BTE and Coelonin at some tested concentrations (Fig. 8). Moreover, PM_2.5_ water or organic extract induced phosphorylation of MAPK family protein ERK, JNK and p38 level which was notably attenuated by BTE or Coelonin pretreatment (Fig. 9). In a word, these results suggest that BTE or Coelonin significantly alleviated the activation of MAPK cascade, and particularly mitigated the NF-κB binding through prevention of PM_2.5_ extracts induced IκBα and NF-κBp65 phosphorylation as well as degradation.

**Fig. 10:**
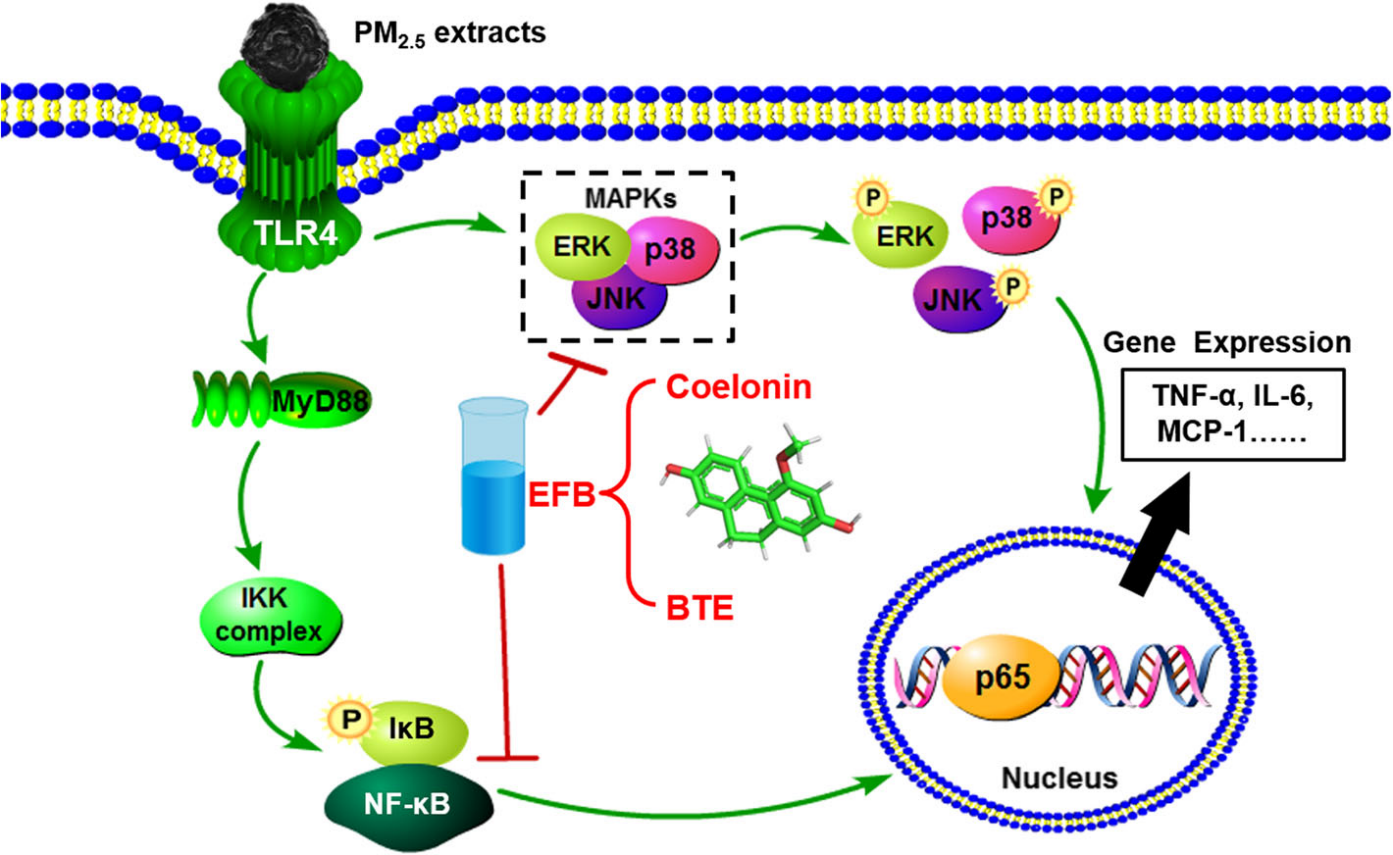
Proposed mechanism of EFB in inhibiting PM_2.5_ extracts activation of NF-κB/MAPK signaling pathways in RAW264.7 cells

## Discussion

Innate immunity is classically viewed as a first line of resistance against pathogens [36]. Macrophages are effector cells of the innate immune system that phagocytose bacteria and secrete both pro-inflammatory and antimicrobial mediators [37]. Exposure to PM_2.5_ activates macrophages, enabling them to initiate defense against, and phagocytosis of, PM_2.5_, which activates a series of transcription factors and promotes cytokine production to inhibit the toxicity of PM_2.5_; however, an excessive inflammatory response can induce cell damage and a systemic inflammatory response [38–40]. IL-6, mainly produced by alveolar epithelial cells, regulates the immune response, participates in inflammatory reactions and anti-infection defense, and is one of the main indicators of early inflammation [41, 42]. TNF-α is a cytokine secreted after stimulation, and it can promote the secretion of cytokines such as IL-6 and IL-8 in cooperation with IL-1, act on alveolar epithelial cells and then further induce increased expression inflammatory factors to initiate an inflammatory response in the lungs [43, 44].

The inflammatory response induced by PM_2.5_ is related to the NF-κB signaling pathway, involving IκBα and IKKβ, and upregulation of this pathway increases the production of inflammatory cytokines [45, 46]. As a nuclear transcription factor, NF-κB is a major signaling protein in inflammatory diseases, and it plays an important role in inflammation, immunity, cell proliferation, and differentiation [47]. The present study showed that IκBα and NF-κB were rapidly elevated in PM_2.5_ extract-treated RAW264.7 cells. BTE and Coelonin attenuated this increased. More and more studies [48, 49] have shown that oxidative stress in tissue and nerve injury-related inflammation is caused by the NF-κB signaling pathway, and the resultant inflammatory factors accelerate the development of systemic diseases, promoting more inflammation and accelerating injury [50]. The cytokine secretion caused by oxidative stress, especially MCP-1 (also known as CCL2P), plays an important role in monocyte recruitment to inflammatory sites. It was previously found that MCP-1 and IL-6 levels in mice exposed to PM_2.5_ increased significantly, indicating that MCP-1 and IL-6 are involved in the inflammatory response to PM_2.5_ [51]. It has also been reported that lung tissue injury induced by PM_2.5_ is related to the activation of the p38/NF-κB signaling pathway, while oxidative stress induced by PM_2.5_ in human vascular endothelial cells increases ICAM-1 and VCAM-1 expression through the ERK/Akt/NF-κB signaling pathway [52].

Macrophages can phagocytose PM_2.5_ and other exogenous substances, resulting in increased reactive oxygen species (ROS) levels, mediated by NADPH on the cell membrane [53]. However, excessive ROS production can increase the permeability of the lysosomal membrane, decreasing lysosomal stability and causing cathepsin B and D release [54]. Cathepsin B can act on mitochondria through Bid and caspase-2, while cathepsin D can act on mitochondria through Bax, resulting in an increase in membrane permeability, the release of cytochrome C, and further inducing the mitochondrial release of ROS [55]. The ROS-MAPK/Akt signaling pathway can improve the airway microenvironment by decreasing the Akt and MAPK phosphorylation induced by ROS and by inhibiting the secretion of the chemokines MCP-1 and IL-8, and intercellular adhesion molecule-1 (ICAM-1) [56].

*B. striata*, a well-known traditional Chinese herb, has been widely used to treat hematemesis, ulcers and skin chapping. Previous studies in our laboratory showed that the chloroform and ethyl acetate extract further separated from the *B. striata* ethanol extract has strong anti-free radical activity, while the chloroform extract also has strong tyrosinase inhibition activity and anti-tumor activity [21] and the chloroform, ethyl acetate, and n-butanol extracts have strong antibacterial activity [19]. It is inferred that *B. striata* and its extracts can interfere with lung injury induced by PM_2.5_. In this study, we explore the effects of BTE or Coelonin on PM_2.5_ water extract or organic extract induced cellular inflammatory injury in cultured the mouse macrophage cell line RAW264.7 and further to investigate the molecular mechanism of anti-inflammatory. An elevated level of proinflammatory cytokines such as TNF-α and IL-6 suggests the establishment of local inflammatory response. Our results showed that the increased level of TNF-α, IL-6 and MCP-1 expression in RAW264.7 cells were attributable to the damaging effects of PM_2.5_ water or organic extract (Figs. 2, 3 and 4). Pretreatment of BTE or Coelonin reduced the secretion of inflammatory cytokines by PM_2.5_ water and organic extract-treated cells, indicating that the components of PM_2.5_ water and organic extracts induce inflammatory cytokine secretion independently of NF-κB, MAPK pathway, and inflammasomes (Fig. 3). The results showed that EFB reduced the mRNA expression of IL-6 and TNF-α, deactivated the NF-κB/MAPK signaling pathways (Fig. 5). Next, we examined EFB decreased expression levels of IκBα and NF-κBp65 phosphorylation in PM_2.5_ extracts induced macrophages. Furthermore, ERK, JNK, and p38 protein phosphorylation levels were assessed by Western blotting (Figs. 8 and 9). Results showed that EFB specifically inhibited the inflammatory response through regulation of NF-κB/MAPK signaling (Fig. 10). These results suggest that Coelonin may be one of the main anti-inflammatory active components of *B. striata* ethanol extract which were in accordance with previously published evidences [25]. Its protective effect on RAW264.7 cells damaged by PM_2.5_ may be related to the inhibition of NF-κB activation and MAPK signals. The secretion of inflammatory cytokine was effectively suppressed by BTE and Coelonin, suggesting that BTE and Coelonin can potentially become effective components of anti-inflammatory medicine. Their specific mechanisms for anti-inflammatory effects are to be further investigated.

## Conclusions

In summary, the present study indicates that PM_2.5_ extracts can induce inflammatory responses and BTE and Coelonin may protect mouse macrophage through anti-inflammatory, possibly via the involvement of NF-κB/MAPK signaling pathways. Based on the protective effects of BTE and Coelonin observed in pretreated cells, the regulation of BTE and Coelonin can be a fascinating approach to prevent/treat macrophage injury in inflammation response. *B. striata*, a well-known traditional Chinese medicine, alleviates PM_2.5_ extract-induced pulmonary inflammation. BTE and Coelonin inhibited the expression of inflammatory cytokines in RAW264.7 cells. Thus, *B. striata* may be useful as a pharmacological agent to protect against PM_2.5_-induced inflammatory disease.

## Supplementary information

**Additional file 1.** Isolation, purification and identification of Coelonin.

## Data Availability

The datasets used and analyzed during this study would be available upon request from the corresponding author.

## Abbreviations

BTE: *Bletilla striata* total ethanol-extract
Coelonin: 2,7-dihydroxy-4-methoxy-9,10-dihydrophenanthrene
DMEM: Dulbecco’s Modified Eagle Medium
DMSO: Dimethyl sulfoxide
EFB: The effective fraction of *Bletilla striata*
ELISA: Enzyme-linked immunosorbent assay
ERK: Extracellular signal-regulated kinase
HPLC: High-performance liquid chromatography
IL-6: Interleukin-6
JNK: c-Jun N-terminal kinase
MAPK: Mitogen-activated protein kinase
MCP-1: Monocyte chemoattractant protein − 1
MTS: (3-(4,5-dimethylthiazol-2-yl)-5-(3-carboxymethoxyphenyl)-2-(4-sulfophenyl)-2H-tetrazolium)
NF-κB: nuclear factor-kappa B
OEP: Organic extracts of PM_2.5_
TNF-α: tumor necrosis factor-α
WEP: Water extracts of PM_2.5_
