# Supplementary material for: Effective fraction of Bletilla striata reduces the inflammatory cytokine production induced by water and organic extracts of airborne fine particulate matter (PM2.5) in vitro
Source: BMC Complement Altern Med. 2019 Dec 16;19:369. doi: 10.1186/s12906-019-2790-3 (PMC6916096; doi:10.1186/s12906-019-2790-3)
Supplement: Supplementary file 1 — Additional file 1. Isolation, purification and identification of Coelonin. [file 12906_2019_2790_MOESM1_ESM.docx]

For the preparation of *Bletilla striata* total ethanol-extract and analyzed by high performance liquid chromatography (HPLC), and the separation, purification and identification of its extract­­---Coelonin, we have published it in the domestic journal (Journal of Chinese Medicinal Materials, title: Spectrum-effect Relationship of Extracts from *Bletilla striata* on Inflammatory Factors Inhibit Activities. Figure 1,2,3 ). I also provide the hydrocarbon spectrum of our group on the Bruker AVANCE III 600 MHz fully digitized superconducting NMR spectrometer after dissolving the compound with Methanol-D4. As shown in the figure below, the hydrocarbon spectrum results are shown as Coelonin (2,7-dihydroxy-4-methoxy-9,10-dihydrophenanthrene) (Figure 4 and 5, the data has not been published yet).


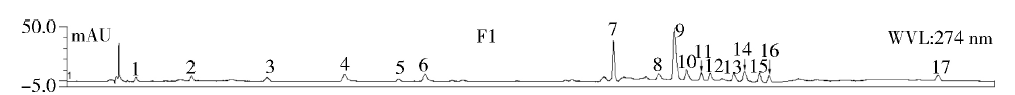


Figure 1. HPLC analysis of *Bletilla striata* total ethanol-extract（BTE）.


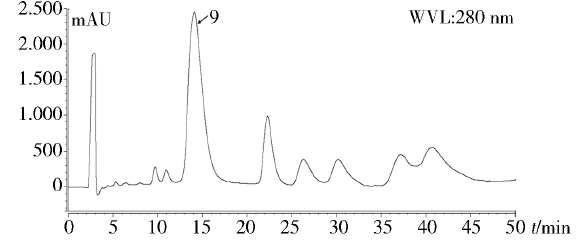


Figure 2. Semi-preparative HPLC of Coelonin, retention time of 12.5~15.5 min.


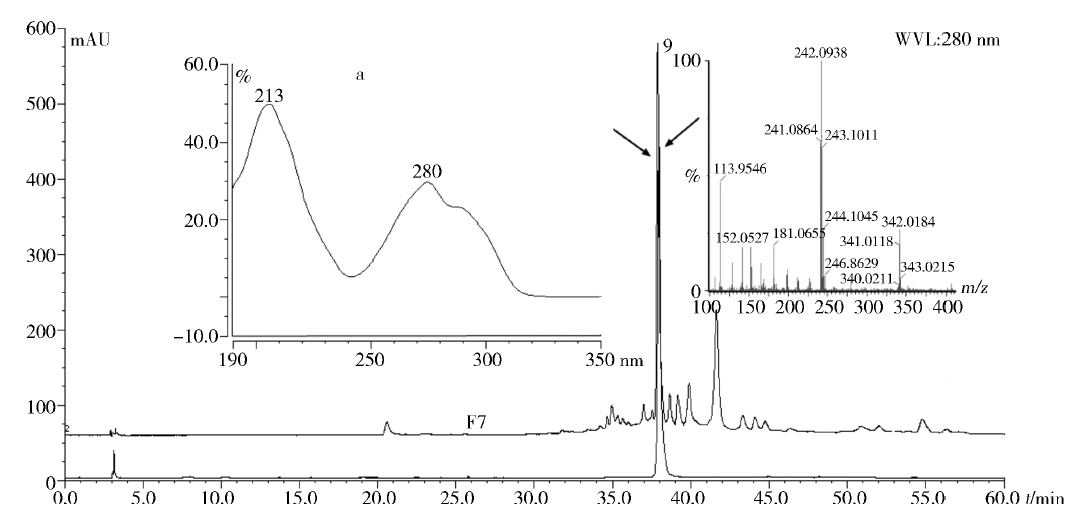


Coelonin mass spectrum of HPLC.

Coelonin ultraviolet spectrum of HPLC.

Figure 3. HPLC purification of Coelonin.


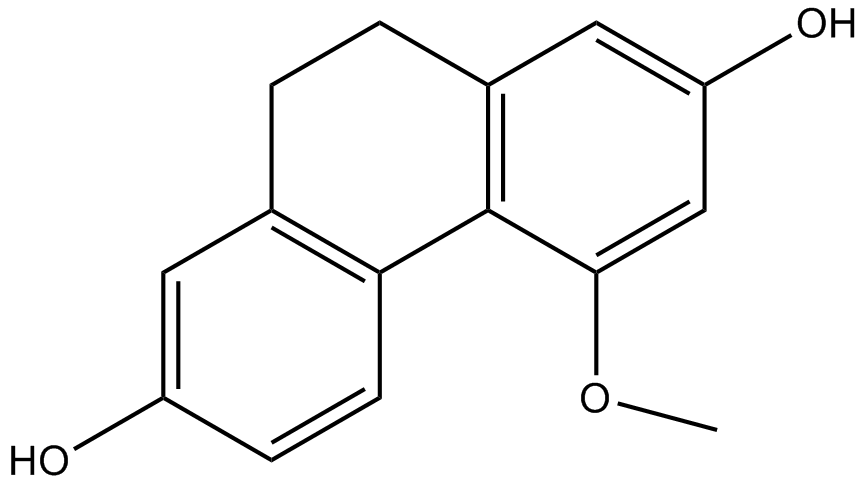

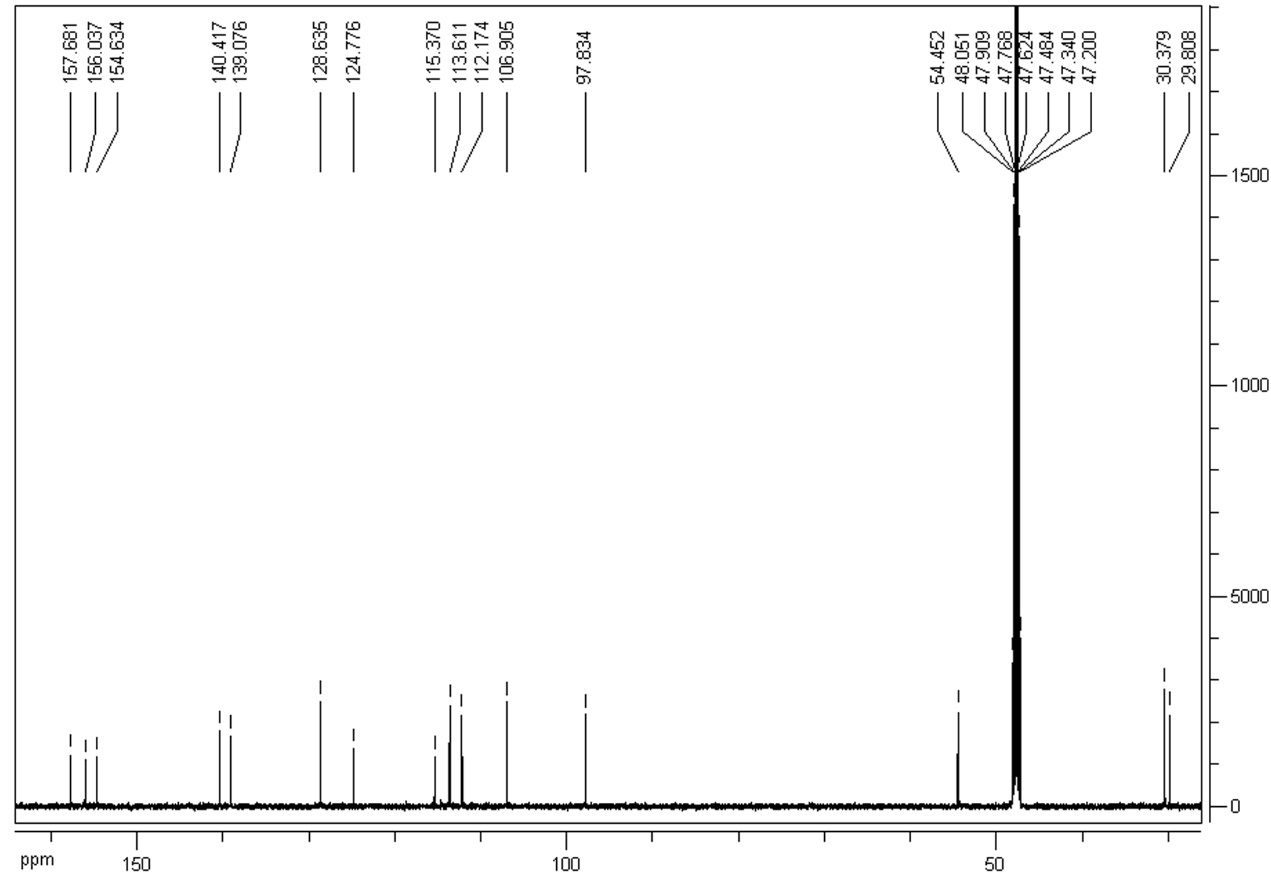


Figure 4. Carbon map of Coelonin.


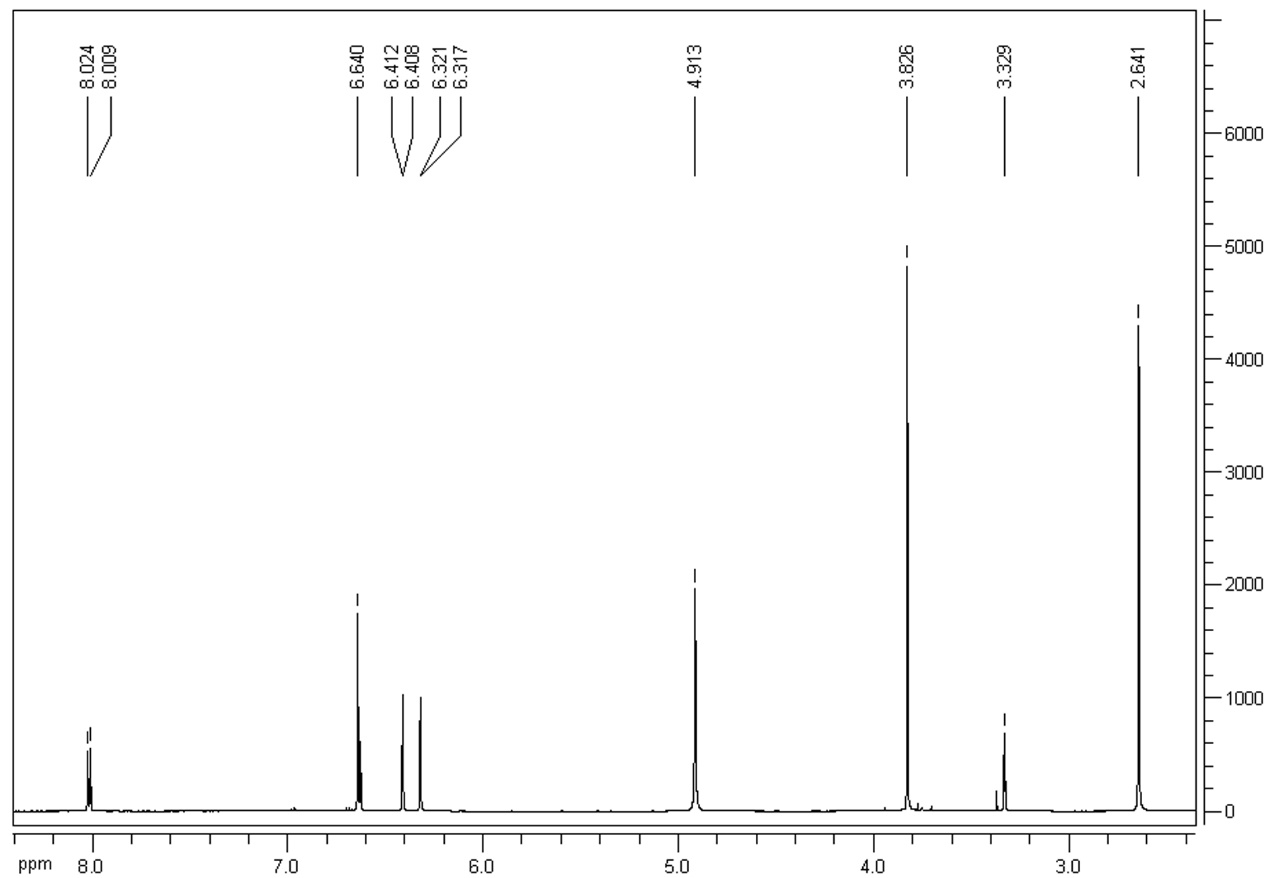


Figure 5. Hydrogen map of Coelonin.

Also refer to our previously published article

1、Jiang FS, Li MY, Wang HY, Ding B, Zhang CC, Ding ZS, Yu XB, Lv GY. Coelonin, an Anti-Inflammation Active Component of *Bletilla striata* and Its Potential Mechanism. Int J Mol Sci. 2019;20.
